# Radical Cure for Hernia

**Published:** 1875-08

**Authors:** 


### RADICAL CURE FOR HERNIA.

Greensville Dowell, M.D., Galveston, thus describes his plan for the radical cure of hernia: The only instruments used are a double, spear-pointed, semi-circular needle, with an eye in each point, silver wire, a piece of cork, soft wood, or a roll of adhesive plaster.

The parts being well shaven, three lines are then drawn with a brush and tincture of iodine, parallel to the direction of the hernia orifice, the centre line being immediately over the internal orifice and passing down to the external orifice, if the hernia be oblique inguinal; in other varieties, immediately over the greatest enlargement of the tumor. The needle is then taken hold of by the left hand at its unthreaded end; then the right hand, with the thumb and forefinger, pulls up the skin and superficial fascia as high as it can be done to the right of the middle line, letting the middle line be just below the point of the thumb. The threaded end is then pushed through the fold held below the point of the thumb and index finger. The fold is then let loose, and the thr aded end taken by its point with the thumb and fingers of the right hand; it is then pulled on until the unthreaded end comes just under the outside line of right side of the operator, and left side of patient. The index finger of the left hand is made to invaginate the integurments as far as possible. and the finger pushed to the right unde- the left tendon of patient, feeling well the wall. The right hand then raises the needle so as to have its point directly over the point of the finger and a little to the outside of it. The needle is then pushed directly down through the tendon into the peritoneal cavity; at this stage, the point of the index finger of the left hand is carried to the right side of the patient, and held under the tendon s; the needle is then moved about to see if it is loose, and turned in its curve so as to carry the curved portion of its point under the invaginated integuments, etc., to about a quarter of an inch of the right tendon; the end is then brought out on the outside line of the patient's right side; this is done by pressing down on the threaded end held by the surgeon's right hand. The index finger of the left hand is then taken out, and the threaded end let go, and the unthreaded end is taken hold of by the right thumb and index finger of the right hand. It is now gently pulled on until the threaded end comes above the tendon. The point threaded is then reversed, and, keeping well down on the tendon, is finally pushed out at the first puncture and pulled entirely out, leaving the two ends of the ligature close together in the same puncture. We have thus put a ligature entirely around the two sides of the rupture with a sufficient portion of the tendon and muscles to give the thread sufficient surface to act on: and now, by pulling on the two ends, the rupture is closed internally, by the replacing of its natural support, and then the ends are tied around a piece of cork or soft wood. If one ligature does not close the opening, so that you can not push the point of your finger under the wire, another wire is put in in the same way. Before tying the first, you must put in enough to completely close the rupture, and they should not be more than a quarter or half an inch apart.

The operation can be performed from either side, but it is best, in inguinal hernia, to start the needle from the side opposite to the ilio-pubic ligament. This enables you to push down the needle by the side of the ligament, when, if you started on the side in the second position of the needle, you may go under the ligament.

This method is simple and easy to perform, and is applicable to all external hernia, direct, scrotal, intestinal, inguinal, frænicular, crural, femoral, umbilical, ventral, epigastric, hypochondriac, lumbar, labial, perineal, and hernia in tunica propria cordæ testis and tunica, tropaia cordæ rotundæ (in canal of Nash). The process is the same, and made with the same needle and silver wire. Of course it is not applicable to internal hernia, as diaphragmatic, obturatic, ischiatic, entrocystic, invaginal, vaginal and rectal, as they can not be reached without resorting to the *direct method*, which ought to be done in all cases of strangulated hernia, when this needle will much facilitate a closure of the incision.

The wires are to be left in from four to seven days, according to the inflammation of the parts. Lotion of sugar of lead morphine are to be applied externally, according to circumstances.—*Trans. Texas Med. Soc.*, 1874.

